# Supercritical Fluid Extract of Spent Coffee Grounds Attenuates Melanogenesis through Downregulation of the PKA, PI3K/Akt, and MAPK Signaling Pathways

**DOI:** 10.1155/2016/5860296

**Published:** 2016-06-07

**Authors:** Huey-Chun Huang, Chien-Mei Wei, Jen-Hung Siao, Tsang-Chi Tsai, Wang-Ping Ko, Kuei-Jen Chang, Choon-Hoon Hii, Tsong-Min Chang

**Affiliations:** ^1^Department of Medical Laboratory Science and Biotechnology, China Medical University, No. 91 Hsueh-Shih Road, Taichung 40402, Taiwan; ^2^Department of Applied Cosmetology and Master Program of Cosmetic Science, Hungkuang University, No. 1018, Section 6, Taiwan Boulevard, Shalu District, Taichung 43302, Taiwan; ^3^O'right Plant Extract R&D Center, No. 18, Gaoping Section, Jhongfong Road, Longtan District, Taoyuan 32544, Taiwan; ^4^Department of Emergency Medicine, Kuang Tien General Hospital, No. 321, Jingguo Road, Dajia District, Taichung 43761, Taiwan

## Abstract

The mode of action of spent coffee grounds supercritical fluid CO_2_ extract (SFE) in melanogenesis has never been reported. In the study, the spent coffee grounds were extracted by the supercritical fluid CO_2_ extraction method; the chemical constituents of the SFE were investigated by gas chromatography-mass spectrometry (GC-MS). The effects of the SFE and its major fatty acid components on melanogenesis were evaluated by mushroom tyrosinase activity assay and determination of intracellular tyrosinase activity and melanin content. The expression level of melanogenesis-related proteins was analyzed by western blotting assay. The results revealed that the SFE of spent coffee grounds (1–10 mg/mL) and its major fatty acids such as linoleic acid and oleic acid (6.25–50 *μ*M) effectively suppressed melanogenesis in the B16F10 murine melanoma cells. Furthermore, the SFE decreased the expression of melanocortin 1 receptor (MC1R), microphthalmia-associated transcription factor (MITF), tyrosinase, tyrosinase-related protein-1 (TRP-1), and tyrosinase-related protein-2 (TRP-2). The SFE also decreased the protein expression levels of p-JNK, p-p38, p-ERK, and p-CREB. Our results revealed that the SFE of spent coffee grounds attenuated melanogenesis in B16F10 cells by downregulation of protein kinase A (PKA), phosphatidylinositol-3-kinase (PI3K/Akt), and mitogen-activated protein kinases (MAPK) signaling pathways, which may be due to linoleic acid and oleic acid.

## 1. Introduction

Melanin is produced and secreted by melanocytes that are distributed in the basal layer of the skin epidermis [1]. Melanin is responsible for skin color and also plays an important critical role in protecting the skin against ultraviolet (UV) light damage. It is reported that accumulation of an excessive level of epidermal melanin resulted in various dermatological disorders. Those skin hyperpigmented syndromes include melasma, postinflammatory melanoderma, age spots, freckles, and sites of actinic damage [2]. Recently, the inhibitors of melanogenesis have been increasingly applied in skin care products for the treatment or prevention of skin hyperpigmented disorders [3]. It is well known that tyrosinase (EC 1.14.18.1) catalyzes the first two steps of melanin synthesis. It first hydroxylates L-tyrosine to L-3, 4-dihydroxyphenylalanine (L-DOPA), and L-DOPA is further oxidized into the corresponding* o*-dopaquinone [4]. There are many factors that participate in the regulation of melanogenesis. For example, the microphthalmia-associated transcription factor (MITF), tyrosinase-related protein-1 (TRP-1), and tyrosinase-related protein-2 (TRP-2) were reported to regulate the production of melanin [5–7]. In addition, the melanocortin 1 receptor (MC1R) also plays a key role in alpha-melanocyte stimulating hormone- (*α*-MSH-) induced melanin synthesis [8]. It is reported that *α*-MSH could elevate cyclic adenine monophosphate (cAMP) level and cAMP is usually used to induce the phosphorylation of cAMP response element-binding protein (CREB) and enhance MITF protein levels [9]. In addition, phosphorylation of mitogen-activated protein kinases (MAPK) and signaling cascades of extracellular responsive kinase (ERK), c-Jun N-terminal kinase (JNK), and p38 also modulate melanin synthesis [10, 11]. Hence, some skin whitening agents can inhibit MITF transcriptional activity by decreasing protein levels of tyrosinase or TRP-1 or TRP-2 through downregulation of MAPK-mediated MITF phosphorylation.

*Coffea arabica* is a plant belonging to the family Rubiaceae. The* Coffea* species are widely distributed in the world. Coffee beans and coffee extract have been used as psychoactive beverages or alternative medicine. It was reported that coffee contains caffeine, several vitamins, minerals, and antioxidants [12]. Caffeine potently blocks an inhibitory neurotransmitter in the brain, leading to a net stimulant effect. In addition, caffeine can improve mood and raises metabolism and increases the oxidation of fatty acids [13]. However, there is relatively little knowledge regarding the modes of action of spent coffee grounds in skin care or dermatology, including the kinds of bioactive components in the spent coffee grounds extract and the effects of the extract or its active components on melanin production [13]. Because of the importance of various compounds present in the coffee waste, the extraction method of the coffee waste appears as an important alternative to increase the aggregated value of the agroindustrial residues. The quality of extracts obtained from a raw material is strongly related to the extraction technique employed, and the quality of the extracts is measured by the chemical profile of the product. Supercritical technology is then a modern technique for extraction that seeks to increase quality by exploiting the selectivity of the process. Hence, the spent coffee grounds were extracted by supercritical technology to get the SFE, and the possible skin care effects of the SFE were further evaluated.

The inhibitory effects of spent coffee grounds SFE on melanogenesis were reported by Sung et al. [14]. However, the action mechanisms of spent coffee grounds for depigmentation still remained to be elucidated. The aim of the current study was to investigate the antimelanogenic activity of the SFE in murine B16F10 melanoma cells. The potential action mechanism of the SFE in melanogenesis was also evaluated by examining the MITF transcription regulators and phosphorylation of regulators of PKA, PI3K/Akt, and MAPK signaling pathways.

## 2. Methods

### 2.1. Chemicals and Reagents

The antibodies were from Santa Cruz Biotech (Santa Cruz, CA, USA), and the ECL reagent was from Millipore (MA, USA). Protein kinase regulators including GF 109203X (classical PKC inhibitor), H89 (cAMP-dependent protein kinase inhibitor; PKA inhibitor), 3-isobutyl-1-methyl-xanthine (IBMX), LY294002 (phosphatidylinositol-3-kinase inhibitor; PI3K inhibitor), PD98059 (MEK 1/2-inhibitor), SB203580 (p38 MAPK-inhibitor), SP600125 (c-Jun N-terminal kinase inhibitor; JNK inhibitor), and U0126 (MEK 1-inhibitor) were from Tocris (Ellisville, Missouri, USA). The chemical reagents were purchased from Sigma-Aldrich Chemical Co. (St. Louis, MS, USA).

### 2.2. Preparation of Spent Coffee Grounds Powder

The spent coffee grounds of* arabica* blends were harvested in 2014 from the coffee shops located in Taoyuan County, Taiwan. The spent coffee grounds were washed completely, exposed to sunlight and air-dried for one day, and then dried at 80°C for 2 h in an oven until the water content of the grounds was less than 5%. The dehydrated coffee grounds were pulverized to a fine powder (#50 mesh) with a committed mill (Retsch Ultra Centrifugal Mill and Sieving Machine, Type ZM1, Haan, Germany). The powder was collected in a sealed glass bottle and stored at 25°C until use.

### 2.3. Supercritical Fluid CO_2_ Extraction (SFE) of Spent Coffee Grounds Powder

The pulverized desiccated spent coffee grounds powder (1000 g) was placed in the extraction vessel (2000 mL) of the supercritical fluid CO_2_ extraction (SFE) apparatus (SFE-400S-2000, Metal Industries Research & Development Centre (MIRDC), Kaohsiung, Taiwan). The extraction was with 10% cosolvent of ethanol in supercritical fluid CO_2_ (flow rate, 45 mL/min) at 2,900 psi (=200 bar) in combination with temperature at 50°C for 2 h. The extracts were evaporated to dryness on a rotary evaporator at 40°C under reduced pressure. The concentrated SFEs were weighed and stored at 4°C. The yield obtained from the extraction was 13–15% (dry weight basis). In the following experiments, the SFEs were redissolved in dimethyl sulfoxide (DMSO) as indicated.

### 2.4. Gas Chromatography (GC) Analysis of Spent Coffee Grounds SFE

The fatty acids in the spent coffee grounds SFE were analyzed using a Thermo GC-MS system (GC-MS Trace DSQ-MASS Spectrometer, MSD 201351, Thermo, Minneapolis, MN, USA). An EquityTM-5 capillary column (Supelco, St. Louis, MO, USA) with 100 m length and 0.25 mm inside diameter with a 0.20 *μ*m thick film was used. The oven temperature gradient was programmed as follows: isothermal heat-treatment in a process at 40°C, followed by a 5°C temperature ramp every minute to 100°C, which was held for 5 min. Subsequently, the temperature was increased 5°C every minute to 250°C and held for 20 min. The carrier gas was helium (1 mL/min). The injection port's and detector's temperatures were 285°C. Ionization of the test sample (1 *μ*L) was performed in the EI mode (70 eV). The linear retention indices for all compounds were determined by coinjection of the spent coffee extract with a solution containing a homologous series of C8–C22 n-alkanes [15]. The compound was identified by retention indices and compared with compounds known from the literature [16]. Their mass spectra were also compared with known, previously obtained compounds or from the Trace DSQ-MASS spectral database (Thermo).

### 2.5. Cell Viability Assay

The B16F10 cells (ATCC CRL-6475, BCRC60031) were obtained from the Bioresource Collection and Research Center (BCRC), Taiwan. The cells were maintained in DMEM (Hyclone, Logan, UT) supplemented with 10% fetal bovine serum and 1% antibiotics at 37°C, 5% CO_2_ in a humidified incubator. The cell viability assay was performed using 3-(4,5-dimethylthiazol-2-yl)-2,5-diphenyltetrazolium bromide (MTT) method [17]. The cells (5 × 10^4^ cells/mL) were exposed to various concentrations of spent coffee grounds SFE (1, 5, and 10 mg/mL) or the same volume of DMSO (as negative control) for 24 h, and the MTT solution was then added to the wells. The insoluble derivative of MTT produced by intracellular dehydrogenase was solubilized with ethanol-DMSO (1 : 1 mixture solution). The absorbance of the wells at 570 nm was read using a microplate reader. Results are expressed as percent viability relative to control and the data are presented as the mean values ± SD from three independent experiments performed in triplicate.

### 2.6. Assay of Mushroom Tyrosinase Activity

The mushroom tyrosinase inhibition experiments were conducted as previously described [18]. In brief, 10 *μ*L of the aqueous solution of mushroom tyrosinase (200 units) was added to a 96-well microplate, for a total volume of a 200 *μ*L mixture containing 5 mM L-DOPA, which was dissolved in 50 mM phosphate buffered saline (PBS) (pH 6.8), spent coffee grounds SFE (1, 5, and 10 mg/mL), or arbutin (2 mM). The assay mixture was incubated at 37°C for 30 min and the absorbance of dopachrome produced was measured at 490 nm. The results are presented as percentages of the control and the data are presented as the mean values ± SD from three independent experiments performed in triplicate.

### 2.7. Measurement of Melanin Content

The intracellular melanin content was measured as described by Tsuboi et al. [19]. The B16F10 melanoma cells (5 × 10^4^ cells/mL) were treated with *α*-MSH (100 nM) for 24 h, and the melanin content was then determined after treatment with either spent coffee grounds SFE (1, 5, and 10 mg/mL) or arbutin (2 mM) for an additional 24 h. After treatment, the cell pellets containing a known number of cells were solubilized in 1 N NaOH at 60°C for 60 min. The melanin content was assayed at 405 nm. The results are presented as percentages of the control and the data are presented as the mean values ± SD from three independent experiments performed in triplicate.

### 2.8. Assay of Intracellular Tyrosinase Activity

The cellular tyrosinase activity was determined as described previously [20]. The B16F10 melanoma cells (5 × 10^4^ cells/mL) were treated with *α*-MSH (100 nM) for 24 h and then with spent coffee grounds SFE (1, 5, and 10 mg/mL) or arbutin (2 mM) for 24 h. After treatments, the cell extracts (100 *μ*L) were mixed with freshly prepared L-DOPA solution (0.1% in PBS) and incubated at 37°C; the absorbance at 490 nm was measured. The results are presented as percentages of the control and the data are presented as the mean values ± SD from three independent experiments performed in triplicate.

### 2.9. Western Blotting Assay

The cells were treated with spent coffee grounds SFE (1, 5, and 10 mg/mL) or arbutin (2 mM), lysed in proteinase inhibitor containing PBS at 4°C for 20 min. Proteins (50 *μ*g) were resolved by SDS-polyacrylamide gel electrophoresis and electrophoretically transferred to a polyvinylidene fluoride (PVDF) filter. The filter was blocked in 5% fat-free milk in PBST buffer (PBS with 0.05% Tween-20) for 1 h. After a brief wash, the filter was incubated overnight at 4°C with several antibodies; these antibodies included anti-MITF (1 : 1000), anti-TRP-1 (1 : 6000), anti-TRP-2 (1 : 1000), anti-MC1R (1 : 500), anti-GAPDH (1 : 1500), anti-tyrosinase (1 : 2000), anti-p-p38 (1 : 500), anti-p38 (1 : 500), anti-p-JNK (1 : 500), anti-JNK (1 : 500), anti-p-ERK (1 : 500), anti-ERK (1 : 500), anti-p-CERB (1 : 500), and anti-CERB (1 : 200). Following incubation, the filter was extensively washed in PBST buffer. Subsequent incubation with goat anti-mouse antibody (1 : 10000) conjugated with horseradish peroxidase was conducted at room temperature for 2 h. The blot was visualized using an ECL reagent. The relative amounts of expressed proteins compared to total GAPDH were analyzed using Multi Gauge 3.0 software (Fuji, Tokyo).

### 2.10. Protein Kinase Regulators Assay

The cells were treated with *α*-MSH (100 nM) for 24 h followed by a 1 h addition of 10 *μ*M of different protein kinase regulators, including GF109203X, H89, IBMX, LY294002, PD98059, SB203580, SP600125, and U0126, respectively. After these treatments, spent coffee grounds SFE (10 mg/mL) and 10 *μ*M of the above kinase regulators were added to the cells and incubated for an additional 23 h, respectively. The melanin contents were assayed as described above. The results are presented as percentages of the control and the data are presented as the mean values ± SD from three independent experiments performed in triplicate.

### 2.11. Statistical Analysis

Statistical analysis of the experimental data points was performed by the ANOVA test, which was used for comparison of measured data using SPSS 12.0 statistical software (SPSS Inc., Chicago, USA). Differences were considered as statistically significant at *p* ≤ 0.05.

## 3. Results

The average amounts of fatty acids in spent coffee grounds SFE were shown in Table 1. The fatty acid constituents in the spent coffee grounds SFE are linoleic acid (43.26%), palmitic acid (35.23%), oleic acid (8.86%), stearic acid (7.15%), arachidic acid (2.68%), *α*-linoleic acid (1.26%), behenic acid (0.52%), gadoleic acid (0.34%), lignoceric acid (0.23%), margaric acid (0.11%), myristic acid (0.083%), tricosanoic acid (0.083%), heneicosanoic acid (0.067%), eicosadienoic acid (0.05%), palmitoleic acid (0.047%), and pentadecanoic acid (0.033%) (Table 1). Unsaturated fatty acids like oleic acid and linoleic acid have been reported to lighten ultraviolet-induced hyperpigmentation of the skin [21]. Hence, it is predicted that linoleic acid and oleic acid in the SFE may contribute to their antimelanogenic activities.

**Figure 1:**
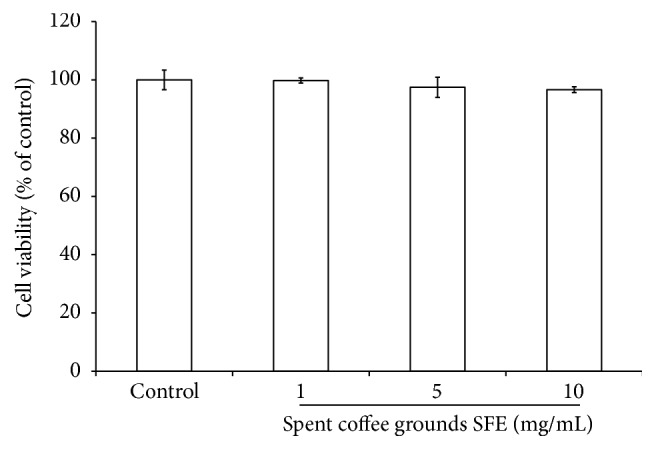
Effect of spent coffee grounds SFE on the proliferation of B16F10 cells. Cell viability was measured by MTT assay method after 24 h incubation. Data are expressed as a percentage of the number of viable cells observed with the control and each column was presented as mean values ± SD from three independent experiments performed in triplicate.

The MTT assay was used to assess the effect of spent coffee grounds SFE on B16F10 cells viability. The cells were treated with various concentrations of the SFE (1, 5, and 10 mg/mL) for 24 h and then MTT assay was performed. After treatment, the spent coffee grounds SFE showed no cytotoxic effect on B16F10 cell viability (Figure 1).

**Figure 2:**
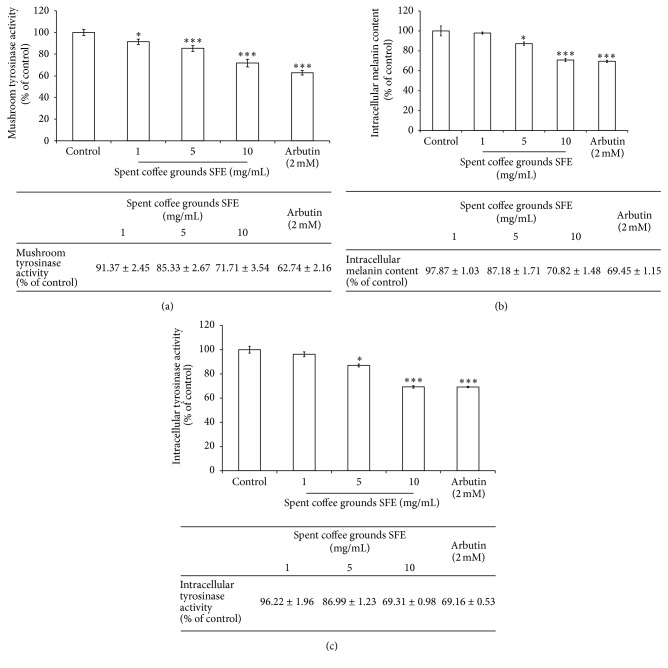
The inhibitory effects of spent coffee grounds SFE on melanogenesis. (a) The effects of spent coffee grounds SFE on mushroom tyrosinase activity. (b) The effects of spent coffee grounds SFE on melanin content in B16F10 cells. (c) The effects of spent coffee grounds SFE on tyrosinase activity in B16F10 cells. The results are presented as percentages of the control, and the data are presented as the mean ± SD of three separate experiments. The values are significantly different compared with the control. ^*∗*^ *p* ≤ 0.05; ^*∗∗∗*^ *p* < 0.001.

The results shown in Figure 2(a) revealed that the remaining mushroom tyrosinase activity was 91.37 ± 2.45%, 85.33 ± 2.67%, and 71.71 ± 3.54% of the control for the 1, 5, and 10 mg/mL of spent coffee grounds SFE treatments, respectively. In addition, the tyrosinase activity was also inhibited by arbutin (2 mM), and the residual enzyme activity was 62.74 ± 2.16% of control (Figure 2(a)).

The results shown in Figure 2(b) indicated that higher concentrations of spent coffee grounds SFE significantly decreased the melanin content in B16F10 melanoma cells. After treatment, the melanin content in the B16F10 cells was 97.87 ± 1.03%, 87.18 ± 1.71%, and 70.82 ± 1.48% for the 1, 5, and 10 mg/mL of spent coffee grounds SFE treatments, respectively. For the positive standard arbutin (2 mM), the intracellular melanin content was 69.45 ± 1.15% of the control (Figure 2(b)). The results indicated that 10 mg/mL of spent coffee grounds SFE showed similar effects as arbutin does.

The B16F10 intracellular tyrosinase activity was 96.22 ± 1.96%, 86.99 ± 1.23%, and 69.31 ± 0.98% for the 1, 5, and 10 mg/mL of spent coffee grounds SFE treatments, respectively. The residual intracellular tyrosinase activity was 69.16 ± 0.53% of control after the cells were treated with arbutin (2 mM) (Figure 2(c)). The results indicated that higher concentration of spent coffee grounds SFE exhibited similar inhibitory effect on *α*-MSH-induced tyrosinase activity in B16F10 cells than arbutin did.

**Figure 3:**
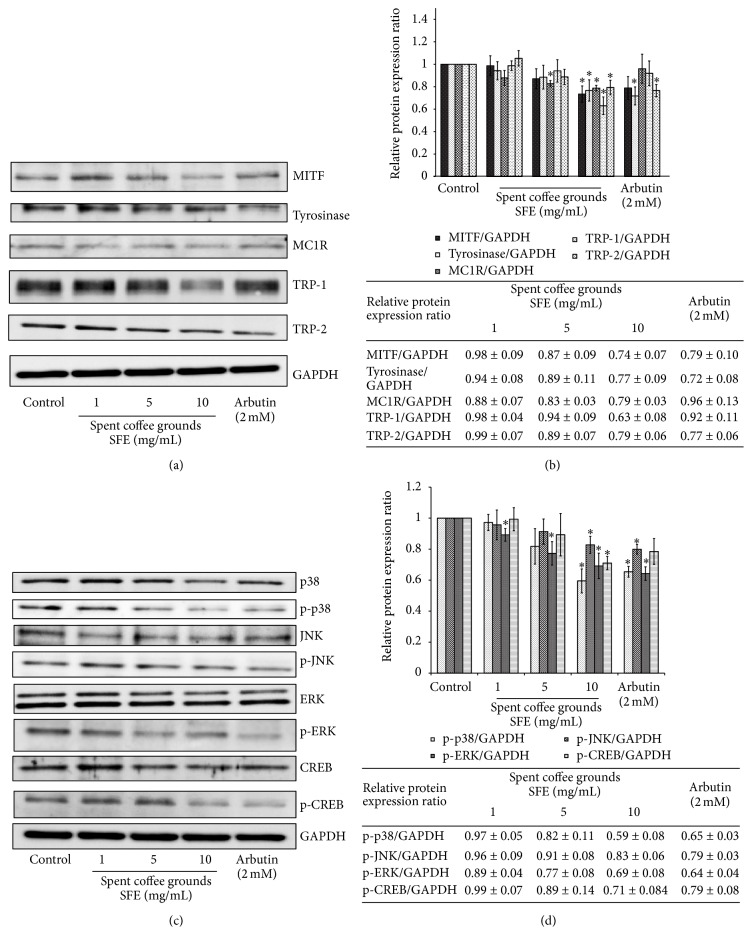
The effects of spent coffee grounds SFE on melanogenesis-related protein expression and signaling pathways. ((a), (c)) Western blotting of cellular proteins in B16F10 cells. ((b), (d)) The relative amounts of MITF, tyrosinase, MC1R, TRP-1, and TRP-2 or phosphorylated proteins (p-p38, p-JNK, p-ERK, and p-CREB) compared to the total GAPDH were calculated and analyzed using Multi Gauge 3.0 software, and the values represented the mean of triplicate experiments ± standard deviations. ^*∗*^ *p* ≤ 0.05.

The expression levels of melanogenesis-related proteins were examined using western blots (Figure 3(a)). The results indicate that 1–10 mg/mL of spent coffee grounds SFE treatment led to a reduced level of MITF, tyrosinase, TRP-1, and TRP-2. The inhibitory effects of the SFE on protein expression were apparent at the concentration of 10 mg/mL. After treatments with the 1, 5, and 10 mg/mL of spent coffee grounds SFE, respectively, the fold changes of protein expression levels were 0.98 ± 0.09, 0.87 ± 0.09, and 0.74 ± 0.07 for MITF; 0.94 ± 0.08, 0.89 ± 0.11, and 0.77 ± 0.09 for tyrosinase; 0.88 ± 0.07, 0.83 ± 0.03, and 0.79 ± 0.03 for MC1R; 0.98 ± 0.04, 0.94 ± 0.09, and 0.63 ± 0.08 for TRP-1; 0.99 ± 0.07, 0.89 ± 0.07, and 0.79 ± 0.06 for TRP-2 (Figure 3(b)).

The protein expression levels of melanogenesis-related signaling proteins were examined using western blots (Figure 3(c)). The results indicate that 1–10 mg/mL of spent coffee grounds SFE treatment led to a reduced level of p-JNK, p-p38, p-ERK, and p-CREB. The inhibitory effects of the SFE on protein expression were apparent at the concentration 10 mg/mL. The fold changes of protein expression levels for p-JNK were 0.96 ± 0.09, 0.91 ± 0.08, and 0.83 ± 0.06; for p-p38 were 0.97 ± 0.05, 0.82 ± 0.11, and 0.59 ± 0.08; for p-ERK were 0.89 ± 0.04, 0.77 ± 0.08, and 0.69 ± 0.08; for p-CREB were 0.99 ± 0.07, 0.89 ± 0.14, and 0.71 ± 0.04 for the 1, 5, and 10 mg/mL of spent coffee grounds SFE treatments, respectively (Figure 3(d)).

**Figure 4:**
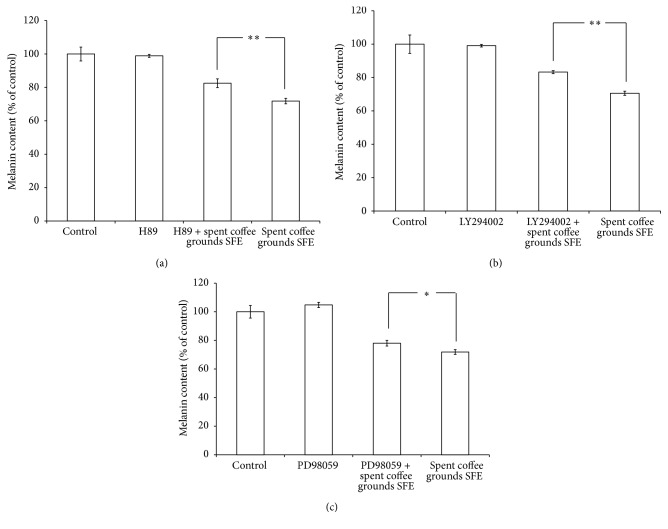
The effects of spent coffee grounds SFE on melanin content in H89 (a), Ly294002 (b), and PD98059 (c) treated B16F10 cells, respectively. The results are represented as percentages of the control, and the data are presented as the mean ± SD of three separate experiments. The values are significantly different compared with the control. ^*∗*^ *p* ≤ 0.05; ^*∗∗*^ *p* < 0.01.

The addition of the SFE to H89 treated B16F10 cells significantly decreased the cellular melanin content, which indicated that PKA-mediated signaling pathway was affected by spent coffee grounds SFE (Figure 4(a)). The results shown in Figure 4(b) revealed that the specific inhibitor of PI3K/Akt, LY294002, attenuated *α*-MSH-stimulated melanin synthesis. These results suggest that spent coffee grounds SFE inhibited melanin synthesis by downregulating PI3K/Akt signaling and, subsequently, decreased melanin synthesis in *α*-MSH-stimulated B16F10 cells. The addition of spent coffee grounds SFE in PD98059 treated B16F10 cells also significantly decreased the cellular melanin content. The results indicate that the ERK-mediated signaling pathway involved in melanin production was affected by spent coffee grounds SFE treatment (Figure 4(c)).

**Figure 5:**
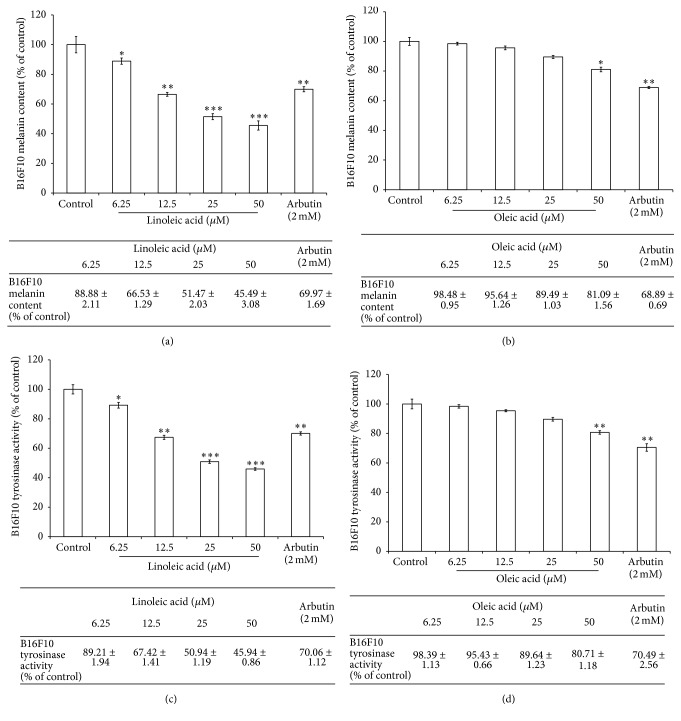
The inhibitory effects of linoleic acid and oleic acid on melanogenesis. ((a), (b)) The effects of linoleic acid (a) and oleic acid (b) on melanin content in B16F10 cells. ((c), (d)) The effects of linoleic acid (c) and oleic acid (d) on tyrosinase activity in B16F10 cells. The results are presented as percentages of the control, and the data are presented as the mean ± SD of three separate experiments. The values are significantly different compared with the control. ^*∗*^ *p*≤ 0.05; ^*∗∗*^ *p* < 0.01; ^*∗∗∗*^ *p* < 0.001.

To further investigate the biological activities of the major components of spent coffee grounds SFE, the potential inhibitory effects of linoleic acid, oleic acid, palmitic acid, and stearic acid on cellular melanin content and tyrosinase activity were investigated. After treatment, the melanin content in the B16F10 cells was 88.88 ± 2.11%, 66.53 ± 1.29%, 51.47 ± 2.03%, and 45.49 ± 3.08% for the 6.25, 12.5, 25, and 50 *μ*M of linoleic acid treatments, respectively (Figure 5(a)). The melanin content in the cells was 98.48 ± 0.95%, 95.64 ± 1.26%, 89.49 ± 1.03%, and 81.09 ± 1.56% for the 6.25, 12.5, 25, and 50 *μ*M of oleic acid treatments, respectively (Figure 5(b)). The results shown in Figures 5(a) and 5(b) indicated that linoleic acid and oleic acid significantly decreased intracellular melanin content. The remaining intracellular tyrosinase activity was 89.21 ± 1.94%, 67.42 ± 1.41%, 50.94 ± 1.19%, and 45.94 ± 0.86% for the 6.25, 12.5, 25, and 50 *μ*M of linoleic acid treatments, respectively (Figure 5(c)). In addition, the intracellular tyrosinase activities were also inhibited by oleic acid. The remaining intracellular tyrosinase activity was 98.39 ± 1.13%, 95.43 ± 0.66%, 89.64 ± 1.23%, and 80.71 ± 1.18% for the 6.25, 12.5, 25, and 50 *μ*M of oleic acid treatments, respectively (Figure 5(d)). However, both palmitic acid and stearic acid showed no inhibitory effects on intracellular tyrosinase activity and melanin content (data not shown). Hence, this study suggests that the antimelanogenic activity of spent coffee grounds SFE was probably due to the inhibitory effects of linoleic acid and oleic acid on intracellular tyrosinase activity and subsequently decreased melanin production.

## 4. Discussion

The MTT assay is a common colorimetric assay to measure the activity of NADH/NADPH-dependent cellular oxidoreductase enzymes that reduce MTT to formazan dyes, giving a purple color. This assay could be used to determine the cytotoxicity of potential medicinal agents and toxic materials, since those agents enhance or inhibit cell viability. The results shown in Figure 1 indicated that the spent coffee grounds SFE showed no cytotoxic effect on B16F10 melanoma cell viability. Thus, we used 1–10 mg/mL of the spent coffee grounds SFE in the following experiments.

Mushroom tyrosinase is widely applied as the target enzyme in screening potential inhibitors of melanin production. It was first found that the dosage range (1–10 mg/mL) of the spent coffee grounds SFE could inhibit the activity of mushroom tyrosinase. The results shown in Figure 2(a) indicated that the spent coffee grounds SFEs show lower inhibitory effect on mushroom tyrosinase activity than arbutin does. Tyrosinase plays an essential role in the melanin synthesis pathway. To evaluate the inhibitory effect of the spent coffee grounds SFE on melanin production, we measured the B16F10 melanin content and intracellular tyrosinase activity after treatment with the extract. The results shown in Figure 2(b) indicated that the spent coffee grounds SFE (10 mg/mL) exhibits a similar inhibitory effect on melanin formation as arbutin does. The results shown in Figure 2(c) were in accordance with the results indicated in Figure 2(b), which means the spent coffee grounds SFE inhibited B16F10 intracellular tyrosinase activity and decreased the melanin content. The data provided evidence that the spent coffee grounds SFE truly downregulates melanin production in the murine melanoma cells. In those intracellular experiments, we used *α*-MSH as a cAMP inducer to stimulate melanin production. *α*-MSH was reported to bind melanocortin 1 receptor (MC1R) and then activate adenylate cyclase, which in turn catalyzes ATP to cAMP and increases the intracellular cAMP level [9]. Further, cAMP-mediated PKA signaling pathway was activated and melanogenesis was enhanced. The results indicated that the spent coffee grounds SFE could inhibit melanin synthesis induced by *α*-MSH mediated intracellular cAMP upregulation.

It was found that binding of the human MC1R by its ligands can activate the cAMP-mediated signaling pathway and regulate melanogenesis of human melanocytes [22]. In mammalian cells, melanin synthesis was also regulated by tyrosinase, TRP-1, and TRP-2 [23]. In addition, MITF is the major transcriptional regulator of the tyrosinase, TRP-1, and TRP-2 genes and is the most important regulator of melanocyte differentiation and melanogenesis [24]. The results shown in Figures 3(a) and 3(b) indicated that the spent coffee grounds SFE decreased the protein expression levels of MITF, tyrosinase, MC1R, TRP-1, and TRP-2 and finally decreased melanin content in the B16F10 cells. The results shown in Figure 3(c) indicated that spent coffee grounds SFE decreased MC1R expression and further suggests that spent coffee grounds SFE inhibited melanogenesis induced via *α*-MSH-mediated intracellular cAMP upregulation. Moreover, the results shown in Figure 4(a) further confirm that spent coffee grounds SFE inhibited cAMP-mediated PKA signaling.

It has been reported that MAPK act to modulate melanin synthesis [25–27]. The MAPK family consists of three types of protein kinases, including extracellular responsive kinase (ERK), c-Jun N-terminal kinase (JNK), and p38 MAPK. It was found that the p38 MAPK can activate the cAMP response element-binding protein (CREB) and then CREB activates MITF expression, which contributes to melanin production [28]. The results in Figure 3(c) provide evidence that spent coffee grounds SFE could inactivate ERK, JNK, p38, and CREB, which in turn inhibits MITF expression (Figure 3(a)). Furthermore, the protein kinase A (PKA) signaling is also reported to be involved in melanin production [29]. The *α*-MSH-mediated elevation of cellular cAMP levels could activate PKA. In turn, activated PKA can activate CREB, leading to the activation of MITF transcriptional activity and resulting in the expressions of melanogenesis-related proteins. Our results shown in Figure 4(a) also suggest that spent coffee grounds SFE inhibits melanin synthesis through downregulation of the PKA pathway. Figure 4(b) revealed that the specific inhibitor of PI3K/Akt, LY294002, attenuated *α*-MSH-stimulated melanin production, which suggested that spent coffee grounds SFE inhibited melanin synthesis through downregulating PI3K/Akt signaling and, subsequently, decreased melanin synthesis in *α*-MSH-stimulated B16F10 cells. Figure 4(c) indicated that the addition of spent coffee grounds SFE in PD98059 treated B16F10 cells also decreased the cellular melanin content, which implied that the ERK-mediated signaling pathway involved in melanin production was affected by spent coffee grounds SFE treatment. The results shown in Figure 5 indicated that linoleic acid and oleic acid may contribute to the inhibitory effects of the spent coffee grounds SFE on melanogenesis. Although we carried out the same experiments for palmitic acid and stearic acid, the two kinds of fatty acids exhibited no inhibitory effects on melanin production in B16F10 cells (data not shown).

The previous study has shown that the SFC from spent coffee exhibited significantly high activities in the inhibition of melanin synthesis and tyrosinase activity [14], which is in accordance with our results. The authors concluded that high fatty acid content did not correspond with the capacity to inhibit melanin synthesis or tyrosinase activity. However, our results have found that the unsaturated fatty acids in the spent coffee grounds SFE play an important role in the inhibition of melanogenesis. The GC-MS analysis results shown in Table 1 revealed that linoleic acid, oleic acid, palmitic acid, and stearic acid were the four major components in the spent coffee grounds SFE. Free fatty acids have been shown to have remarkable regulatory effects on melanogenesis in cultured B16F10 murine melanoma cells. Unsaturated fatty acids, such as oleic acid (C18:1), linoleic acid (C18:2), or *α*-linolenic acid (C18:3), decreased melanin synthesis and tyrosinase activity, while saturated fatty acids, such as palmitic acid (C16:0) or stearic acid (C18:0), increased it [21, 30, 31]. Those reports supported our results shown in Figure 5 that oleic acid and linoleic acid in the spent coffee grounds SFE contributed to the inhibition of melanogenesis in the B16F10 cells. Interestingly, it has been reported that topical application of linoleic acid lightened UV-stimulated hyperpigmented skin of experimental guinea pig [9], which further supported our proposal that linoleic acid existing in the SFE might play an important role in the inhibitory effects on melanogenesis in the melanoma cells. In addition, the regulatory effects of fatty acids on melanin production are probably through proteolytic degradation of tyrosinase [32]. Our results indicated that spent coffee grounds SFE inhibited melanogenesis in B16F10 cells by downregulation of both mitogen-activated protein kinases (MAPK) and protein kinase A (PKA) signaling pathways. Hence, the spent coffee grounds SFE could be used as an effective skin whitening agent.

## 5. Conclusion

This is the first report on the action mechanisms of the inhibitory effect of spent coffee grounds SFE on melanin biosynthesis. The present study concluded that spent coffee grounds SFE inhibits melanin synthesis in B16F10 melanoma cells by downregulation of protein kinase A (PKA), phosphatidylinositol-3-kinase (PI3K/Akt), and mitogen-activated protein kinases (MAPK) signaling pathways. Hence, the spent coffee grounds SFE could be used as a novel dermatological antimelanogenesis agent in skin care products.

## Figures and Tables

**Table 1:** Fatty acid composition of spent coffee grounds SFE.

Fatty acid	Carbon number	Content (%)
Myristic acid	C14:0	0.083 ± 0.0047
Pentadecanoic acid	C15:0	0.033 ± 0.0047
Palmitic acid-2	C16:0	35.23 ± 0.2628
Palmitoleic acid	C16:1	0.047 ± 0.0170
Margaric acid-9	C17:0	0.11 ± 00142
Stearic acid-4	C18:0	7.15 ± 0.0712
Oleic acid-3	C18:1	8.8567 ± 0.066
Linoleic acid-1	C18:2	43.2567 ± 0.296
*α*-Linoleic acid	C18:3	1.2567 ± 0.017
Arachidic acid-5	C20:0	2.68 ± 0.0589
Gadoleic acid-7	C20:1	0.3367 ± 0.0047
Eicosadienoic acid	C20:2	0.05 ± 0.0023
Heneicosanoic acid	C21:0	0.0667 ± 0.0047
Behenic acid-6	C22:0	0.52 ± 0.0356
Tricosanoic acid	C23:0	0.083 ± 0.0047
Lignoceric acid-8	C24:0	0.23 ± 0.0163
